# Autism Spectrum disorders (ASD) in South Asia: a systematic review

**DOI:** 10.1186/s12888-017-1440-x

**Published:** 2017-08-01

**Authors:** Mohammad Didar Hossain, Helal Uddin Ahmed, M M Jalal Uddin, Waziul Alam Chowdhury, Mohd S Iqbal, Razin Iqbal Kabir, Imran Ahmed Chowdhury, Afzal Aftab, Pran Gopal Datta, Golam Rabbani, Saima Wazed Hossain, Malabika Sarker

**Affiliations:** 1 James P. Grant School of Public Health, BRAC University, 68 Shahid Tajuddin Ahmed Sharani, Dhaka, Bangladesh; 2 Foundation for Advancement of Innovations in Technology and Health (faith), Bangladesh, Iqbal Road, Mohammadpur, Dhaka, 1207 Bangladesh; 3 National Institute of Mental Health, Bangladesh (NIMH,B), Sher-E-Bangla Nagar, Dhaka, 1207 Bangladesh; 4 National Institute of Neurosciences & Hospital, Bangladesh (NINS,B), Sher-E-Bangla Nagar, Dhaka, 1207 Bangladesh; 5 Bangladesh Association of psychiatrists, National Institute of Mental Health, Bangladesh (NIMH,B), Sher-E-Bangla Nagar, Dhaka, 1207 Bangladesh; 6 Nutrition and Clinical Services Division, International Centre for Diarrhoeal Disease Research, Bangladesh (icddr,b), 68 Shahid Tajuddin Ahmed Sharani, Mohakhali, Dhaka, 1212 Bangladesh; 7 Shuchona Foundation, Bangabandhu Memorial Trust Building, 2nd floor, 8 Rd No 11, Dhaka, 1209 Bangladesh; 8 Bangabandhu Sheikh Mujib Medical University (BSMMU), Dhaka, 1000 Bangladesh; 9 Neuro-Developmental Disability Protection Trust, Department of Social Services Building, Agargaon, Sher-e-Bangla Nagar, Dhaka, 1207 Bangladesh; 10 Expert Advisory Panel on Mental Health; Global Autism Advocate, World Health Organization, Dhaka, Bangladesh; 11 National Advisory Committee for Autism and Neurodevelopmental Disorders, Ministry of Health and Family Welfare, Government of Bangladesh, Dhaka, Bangladesh; 12 Institute of Public Health, University of Heidelberg, Im Neuenheimer Feld, Heidelberg, 69120 Germany

**Keywords:** Autism spectrum disorders, Autism, South Asia, Prevalence, Bangladesh, India, Pakistan, Nepal, Sri Lanka, Bhutan, Maldives, Afghanistan

## Abstract

**Background:**

Autism spectrum disorders (ASD) are a group of complex neurodevelopmental disorders. The prevalence of ASD in many South Asian countries is still unknown. The aim of this study was to systematically review available epidemiological studies of ASD in this region to identify gaps in our current knowledge.

**Methods:**

We searched, collected and evaluated articles published between January 1962 and July 2016 which reported the prevalence of ASD in eight South Asian countries. The search was conducted in line with the PRISMA guidelines.

**Results:**

We identified six articles from Bangladesh, India, and Sri Lanka which met our predefined inclusion criteria. The reported prevalence of ASD in South Asia ranged from 0.09% in India to 1.07% in Sri Lanka that indicates up to one in 93 children have ASD in this region. Alarmingly high prevalence (3%) was reported in Dhaka city. Study sample sizes ranged from 374 in Sri Lanka to 18,480 in India. The age range varied between 1 and 30 years. No studies were found which reported the prevalence of ASD in Pakistan, Nepal, Bhutan, Maldives and Afghanistan. This review identifies methodological differences in case definition, screening instruments and diagnostic criteria among reported three countries which make it very difficult to compare the studies.

**Conclusions:**

Our study is an attempt at understanding the scale of the problem and scarcity of information regarding ASD in the South Asia. This study will contribute to the evidence base needed to design further research and make policy decisions on addressing this issue in this region. Knowing the prevalence of ASD in South Asia is vital to ensure the effective allocation of resources and services.

## Background

Autism spectrum disorders (ASD) are a group of neurodevelopmental conditions characterized by social and communication deficits, stereotyped interests and repetitive behaviors [1]. Approximately one in 68 children are identified with ASD according to estimates from Centers for Disease Control and Prevention (CDC)'s Autism and Developmental Disabilities Monitoring (ADDM) Network [2]. Systematic reviews around the world estimated different prevalence rates of ASD [3–6]. Williams et al. [3] estimated global ASD prevalence of 20 per 10,000 with high degree of heterogeneity among studies. Elsabbagh et al. [5] reported a global median ASD prevalence of 62 cases per 10,000 population which translates to one child out of 160. Baxter et al. [4] estimated prevalence of 7.6 per 1000 or one in 132 persons worldwide. In Asia, Sun and Allison conducted a systematic review in six Asian countries that excluded South Asian countries revealed prevalence of ASD from 1980 to present was 14.8 per 10,000 [6].

In Asia, the South Asia region represents more than 20% of the world’s population, yet the prevalence of ASD in this part of the world is still largely unknown. The region represents eight countries including Bangladesh, India, Pakistan, Nepal, Sri Lanka, Bhutan, Maldives, and Afghanistan [7]. The present study systematically reviews the prevalence of ASD in South Asia and identifies gaps in our current knowledge. Lack of significant authorship and research output for low and middle income countries (LMICs) stunts the development of evidence-based health policies and practice in LMICs [8]. This review aims to provide information on the epidemiology of ASD which will assist health professionals and policy makers in prioritizing relevant research and services in South Asia.

## Methods

### Case definition of ASD

In this review, we used the Diagnostic and Statistical Manual of Mental Disorders, 4th Edition (DSM-IV) [1] definition of ASD where ASD comprise of autistic disorder (299.0); Asperger’s disorder (299.8); pervasive developmental disorders not otherwise specified, including atypical autism (PDD-NOS) (299.8); Rett’s disorder (299.8); and childhood disintegrative disorder (299.1). In DSM-IV, ASDs are grouped as Pervasive Developmental Disorders (PDD), and are characterized by: ‘severe and pervasive impairment in several areas of development: reciprocal social interaction skills, communication skills, or the presence of stereotyped behavior, interests, and activities’ [1].

The Diagnostic and Statistical Manual of Mental Disorders, Fifth Edition (DSM-V) is the 2013 update to the American Psychiatric Association’s classification and diagnostic tool. None of the studies adapted DSM-V during our literature search. As a result, we did not follow ASDs as a spectrum disorder with hierarchical levels of severity as described in DSM-V [9]. The conditions included under the ASD umbrella in DSM-IV [1] vary slightly from the 10th edition of the International Classification of Diseases (ICD-10) [10].

### Search strategy

The search strategy followed the Preferred Reporting Items for Systematic Reviews and Meta-Analyses (PRISMA) checklist and adhered to PRISMA guidelines/methodology [11]. Three electronic databases were searched (PubMed, Medline, and EMBASE) and we identified relevant facility and community based literature using the following databases: Bangladesh Journals Online (BanglaJOL), Indian Journals (IndianJournals.com), Pakistani Medical Journals and Drugs Database (PakMediNet), Sri Lanka Journals Online (SLJOL), Nepal Journals Online (NepJOL), and also searched journals from Bhutan, Maldives and Afghanistan up to the year July, 2016 (searched 31/07/2016; years-1962–2016). The search then extended to a manual search of reference lists for review articles, reports, editorials, and resource texts. An online search of government, university and non-government websites to identify further non-peer-reviewed data sources was also carried out.

The search terms included “Autism Spectrum Disorders” OR “Autism” OR “Autistic disorder” OR “Asperger syndrome” OR “Rett’s syndrome” OR “Childhood disintegrative disorder” OR “Non-specific pervasive disorder” AND “Prevalence” OR “Epidemiology”. A further search was conducted for each country in South Asia one by one with the search terms listed above. We carried out a manual search of the reference lists of these studies to identify additional articles. Additionally, paper versions were acquired for local journals that could not be accessed online. Citations were managed using EndNote version X7.2.1.

### Inclusion and exclusion criteria

We included articles based on following criteria adapted from Wing [12]; (i) Primary research (ii) Quantitative data collected from cross-sectional studies or first phase of a longitudinal study on ASD (iii) Geographically defined population (iv) Defined diagnostic criteria stated for autism or autism spectrum disorders (v) Includes individuals under 18 years old (vi) Reported on human participants (vii) Published between 1962 (earliest listed publication) [13] and July, 2016 (latest publication obtained) [14] (viii) Initial selection in a wide range of children in the general population (ix) Final identification of cases based on clinical or other diagnostic assessment of selected children (x) Peer reviewed papers or conference proceedings and (xi) Prevalence data. We excluded articles which were based on: (i) qualitative studies, and (ii) those published as theses/ dissertations.

### Quality assessment

Initially, two authors (MDH & HUA) screened and evaluated each article independently to decide on its inclusion or exclusion. Articles were further assessed by other four authors AA, MSI, RIK and IAC for (i) the appropriateness and clarity of the research question/objectives/ aims (y/n) and the study design chosen (y/n), (ii) adequate description of study location (y/n), sample/ participants (y/n), data collection methods (y/n), context of collection and quantitative outcome data presented (y/n) and (iii) adequacy of measurement and appropriateness of statistical analysis (e.g. the odds ratio, *p* values and confidence interval) (y/n). SWH, PGD, GR, WAC, MMJU and MS critically reviewed and provided suggestions on the review process. For each article found, titles and abstracts were initially examined to determine whether the selection criteria were met. If an article failed to meet these criteria, the full text article was not retrieved and was excluded. In case of any disagreement on quality assessment checklist, four authors (MDH, HUA, AA and MS) discussed together and reached an agreement about inclusion or exclusion of that particular article.

This study formed two groups. Those that assessed the prevalence of autism, or autistic disorder, known here as ‘typical autism’; and those that assessed the prevalence of autism spectrum disorders (ASD) or all pervasive developmental disorders, known here as ‘all ASD’. The quality of the reports of the included studies was assessed using STROBE (Strengthening the Reporting of Observational Studies in Epidemiology) guidelines, a checklist of 22 items [15].

### Tabulation

**Fig. 1:**
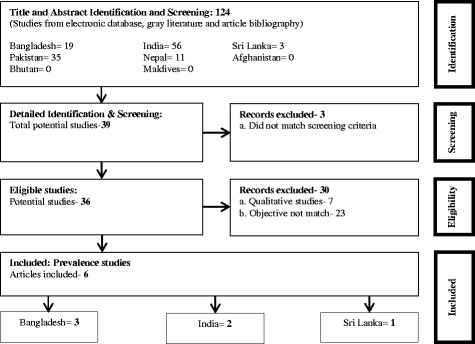
An adapted PRISMA flow diagram of the literature selection process for inclusion in the systematic review [48]

After full examination, we categorized the articles, extracted data from each study and tabulated by (i) first author, (ii) year of publication, (iii) country, (iv) region and place of study (community and facility based, rural and urban), (v) study method: “prevalence/cross-sectional study, case control, cohort, specific population survey and trials”, (vi) screening strategy and information source, (vii) diagnostic criteria and strategy, (viii) age at diagnosis, (ix) size of population, (x) reported prevalence of ASD. If manuscripts contained several analyses, data were extracted only on those analyses that met the inclusion criteria. A record of all the excluded studies and the reasons for exclusion was documented. The selection process of the articles is displayed in Fig. 1.

## Results

### Selection of literature

Through the initial search of databases, we identified 124 articles where 19 articles were from Bangladesh, 56 articles from India, 35 articles from Pakistan, 11 articles from Nepal, 3 articles from Sri Lanka and no articles from Bhutan, Maldives or Afghanistan. After exclusion through comparison of titles and abstracts against inclusion criteria, we excluded 85 articles as they were deemed not relevant to the review and 39 papers were identified for detailed examination. We excluded prospective studies presenting the natural course of the disorder or any rigorously controlled study of any intervention. Out of the remaining 39 articles, 3 failed to meet the screening criteria and full text of the remaining 36 articles were further reviewed and checked for eligibility which resulted in further exclusion of another 30 articles, 7 of which were due to qualitative in nature and 23 which did not fulfill the required methodological criteria. Finally, 6 studies met the inclusion criteria for the review (Fig. 1).

### Prevalence of ASD in South Asia

Table 1 presents a summary of the six community based studies that reported the prevalence of ASD; three studies from Bangladesh [16–18], two from India [14, 19], and one from Sri Lanka [20]. However, there was a wide variation of screening and diagnostic instruments among the six studies. Only two Bangladeshi studies [17, 18] discussed the generalizability of their findings along with the study limitations. Four studies investigated both rural and urban prevalence of ASD. The remaining two studies were limited to semi urban context [14, 20]. Study sample sizes ranged from 374 to 18,480 across these three countries.

**Table 1:** Summary of epidemiological studies on ASD in South Asia

Author’s, publication year and reference	Country	Setting	Place of the study	n (sample size)	Age ranges	Gender	Outcome measures instruments	Prevalence
Mullick & Goodman, 2005 [16]	Bangladesh	Rural & urban	Community	922	5–10y	M & F	SDQ DAWBA	0.2%
Rabbani et al., 2009 [17]	Bangladesh	Rural & urban	Community	3564	5-17y	M & F	RQC	0.84%
NCDC, 2013 [18]	Bangladesh	Rural & urban	Community	7280	0-9y	M & F	DSQ (for 0- < 2 y) TQP (for 2–9 y) ADOS	0.15%
Raina et al., 2015 [19]	India	Rural, urban and tribal	Community	11,000	1–10y	M & F	ISAA	0.09%
Poovathinal et al., 2016 [14]	India	Semi-urban	Community	18,480	1–30 y	M & F	DSM-IV-TR	0.23%
Perera et al., 2009 [20]	Sri Lanka	Semi-urban	Community	374	18-24 m	M & F	M-CHAT	1.07%

*SDQ* Strengths and Difficulties Questionnaire, *DAWBA* Development and Well-Being Assessment, *RQC* Reporting Questionnaire for Children, *DSQ* The Developmental Screening Questionnaire, *TQP* Ten Questions Plus, *ADOS* Autism Diagnostic Observation Schedule, *ISAA* Indian Scale for Assessment of Autism, *DSM-IV-TR* Diagnostic and Statistical Manual of Mental Disorders, Fourth Edition, Text Revision, *M-CHAT* Modified Checklist for Autism in Toddlers, *m* Month, *y* Years, *M & F* Both male and female

In Bangladesh, reported prevalence of autism in children was 0.2% [16], 0.84% [17] and 0.15% [18]. In India, two community based prevalence studies reported 0.09% [19] and 0.23% [14] respectively. In Sri Lanka, only one community based study reported children aged 18–24 months were initially screened for autism, using ‘Red Flag’ criteria, 4 (1.07%) were finally diagnosed using DSM-IV criteria [20]. Among South Asian countries, Sri Lanka reported highest prevalence. Due to the absence of studies from other five South Asian countries (Pakistan, Nepal, Bhutan, Maldives, and Afghanistan) the prevalence of autism in these countries could not be determined.

We found eight different screening and diagnostic tools including Strengths and Difficulties Questionnaire (SDQ), Development and Well-Being Assessment (DAWBA), Reporting Questionnaire for Children (RQC), Developmental Screening Questionnaire (DSQ; for 0- < 2 y), The Ten Questions Plus (TQP; for 2–9 years), Autism Diagnostic Observation Schedule (ADOS), Indian Scale for Assessment of Autism (ISAA) and Modified Checklist for Autism in Toddlers (M-CHAT).

## Discussion

This paper reports prevalence of ASDs for the first time in South Asia. Epidemiological research on the prevalence of ASD has been conducted by only three countries. The available studies reported prevalence of ASD in South Asia ranging from 0.09% in India to 1.07% in Sri Lanka. This data indicates up to one in 93 children have ASD in this region.

The prevalence of ASD ranged from 0.15–0.8% in Bangladesh. In Dhaka, the capital of Bangladesh, prevalence was much higher (3%) [18]. One explanation for this higher prevalence in urban area may be mental health services are concentrated around institutions and no service is available at the primary health care (PHC) level [21]. In the urban hospital, only 12 children were diagnosed with autism in the year 2001 with this number increasing to 105 children in 2009 [22]. Autism related reported cases are increasing due to increased rate of incidence, awareness amongst parents and capability to diagnose the problem [22]. Bangladesh is a lower middle income country with a three leveled health care delivery system [23]. The health system in Bangladesh is pluralistic [24] and a significant proportion of rural people have faith in traditional healers [25, 26]. These factors may act as barriers to proper management of mental disorders [21]. In addition, appropriate care is often missing both at district and sub-district health facilities due to lack of trained professionals [27]. Early detection and early intervention are key to reducing the burden associated with these disorders [18].

In India, prevalence of ASD was unknown until early 2015. ASD research in India is still limited to largely clinic-based case reports, case series, retrospective chart reviews, qualitative studies and treatment studies. These studies did not meet our inclusion criteria as they cannot be considered representative of the general population. Authors of a recent case series reported that autism is not uncommon in India [28, 29]. Its diagnosis is frequently missed as there is tremendous lack of awareness and knowledge about the disorder among health professionals [28, 30]. The first reported prevalence data from India represented preliminary findings, based on a mid-term report submitted to the funding agency [19]. The reported prevalence among their studied pediatric population (1–10 years of age) would be lower if they consider higher cut off score as the ISAA tool has suboptimal validity in 3–9-year-old children [31]. Recent reported prevalence data from the south Indian state of Kerala [14] was higher than previously reported study [19]. This increase though in slow pace indicate the alarming increase in the prevalence of ASD in India. One recent study evaluated the ISAA in relation to the Childhood Autism Rating Scale (CARS) and the Developmental Disability- Children Global Assessment Scale (DD-CGAS) [32]. Another prevalence study that was excluded from this review was limited to siblings of children with ASD and almost equal in male and female siblings [33]. Nationwide study of the prevalence of neurodevelopmental disorders in India has begun and is being carried out by members of the International Clinical Epidemiology Network (INCLEN) including 2–9 years aged children per cluster across five sites (rural, urban, hilly, coastal and tribal) [34] by expanding on the well-known “10 Questions” screen from Bangladesh [35]. It is estimated that there are approximately 1.7–2 million children with Autism Spectrum Disorders in India [36]. Case reports and qualitative studies in India have shown that most diagnosed cases belong to middle-class families. Upper class families do not frequent public health centers to treat autistic children, and families from low socioeconomic strata do not access such facilities unless the child is acutely ill [28, 37, 38].

Perera et al. from Sri Lanka reported the prevalence of 10.7 per 1000 (1.07% or one in 93) [20]. It is relatively higher than the rest of this region, however, consistent with the prevalence rates in the Western world. It is not possible to compare and comment on regional differences in the absence of other studies for comparison. This reported prevalence study was not representative of the whole island. Poor knowledge about autism and lack of collaboration is common. Sri Lanka does not have the necessary infrastructure or trainers to provide intervention for these children.

In Pakistan, prevalence of ASD was limited to either hospital based or had been done in children with autism in special schools, and therefore difficult to generalize the results to other settings [39–44]. There is no reliable estimate of ASD in Nepal as there is lack of awareness amongst people and diagnosis process is poor [45]. Notably, the Government of Nepal has implemented the National Policy and Action plan for Disability-2006 and categorized including autism [46]. Since there is no community based ASD prevalence data in these countries, it is difficult to allocate adequate financial, infrastructural and human resources to address the issue.

The 67th World Health Assembly adopted a resolution that sets out a clear set of actions to facilitate comprehensive inter-sectoral response to the needs of persons with ASD and other developmental disorders in all high, middle and low income countries (Agenda item 13.4, WHA 67.8) [47]. The United Nations General Assembly unanimously declared 2 April as World Autism Awareness Day (A/RES/62/139) to highlight the need to help improve the quality of life of children and adults, who are affected by autism, so they can lead full and meaningful lives.

### Critique of methods

Methodological differences in case definition, screening instruments and diagnostic criteria were evident between our reported countries which makes it very difficult to compare the studies. For example: M-CHAT was used for preliminary screening in study conducted in Sri Lanka [20]. Although it has reasonable reliability and validity across different cultural and ethnic groups, use of this tool as Level II screening tool restricted the age limits of the population to 18–24 months. Due to the different screening and diagnostic tools as well as study objectives of our included studies no uniform age groups were followed. Since ASD prevalence rates may differ across age groups, it is difficult to compare the prevalence rates. Moreover, each tool is subject to its unique sensitivity and specificity to detect cases which again made comparison difficult.

Since only six studies met the inclusion criteria, there might be selection bias and publication bias. The different diagnostic procedures employed within each study might have produced misclassification bias. The targeted population size varied across studies which make the comparison between studies difficult. These studies only covered three countries in South Asia which may lack generalizability across this region as a whole and inadequate data limit conducting meta-analysis. The reported prevalence is only generalizable for the specific age group.

## Conclusion

Our study aimed at systematically reviewing available prevalence surveys of ASD in South Asia. The review is a useful contribution in identifying evidence gap and has important implications for government and NGOs working in this sector. This review identified only a limited number of studies on ASD conducted in this part of the world that limit our conclusion despite the alarming increase in the prevalence of ASD in recent years. To know the extent of ASD as a public health problem, there is an urgent need for all countries of this region to conduct well-designed epidemiological research using uniform and appropriate tools. Knowing the prevalence could help choosing screening and diagnostic tools that are applicable, culturally acceptable, and cost-effective to identify individuals who can benefit the most from early diagnosis and intervention.

## Abbreviations

AD: Autistic Disorder
ADDM: Autism and Developmental Disabilities Monitoring Network
ADOS: Autism Diagnostic Observation Schedule
ASD: Autism Spectrum Disorders
BanglaJOL: Bangladesh Journals Online
BSMMU: Bangabandhu Sheikh Mujib Medical University
CARS: Childhood Autism Rating Scale
CDC: Centers for Disease Control and Prevention
DAWBA: Development and Well-Being Assessment
DD-CGAS: Developmental Disability- Children Global Assessment Scale
DSM-IV: Diagnostic and Statistical Manual of Mental Disorders, Fourth Edition
DSM-V: Diagnostic and Statistical Manual of Mental Disorders, Fifth Edition
DSQ: The Developmental Screening Questionnaire
EMBASE: Excerpta Medica Database
ICD-10: International Classification of Diseases
INCLEN: International Clinical Epidemiology Network
ISAA: Indian Scale for Assessment of Autism
LMIC: Low and middle income countries
M-CHAT: Modified Checklist for Autism in Toddlers
NCDC: Non Communicable Diseases Control
NepJOL: Nepal Journals Online
NGO: Non-Governmental Organization
NIMH,B: National Institute of Mental Health, Bangladesh
NINS,B: National Institute of Neurosciences & Hospital, Bangladesh
PakMediNet: Pakistani Medical Journals and Drugs Database
PDD-NOS: Pervasive Developmental Disorders Not Otherwise Specified
PHC: Primary Health Care
PRISMA: Preferred Reporting Items for Systematic Reviews and Meta-Analyses
RQC: Reporting Questionnaire for Children
SDQ: Strengths and Difficulties Questionnaire
SJOL: Sri Lanka Journals Online
STROBE: Strengthening the Reporting of Observational Studies in Epidemiology
TQP: Ten Questions Plus
WHA: World Health Assembly
